# Social and Poor Law Problems

**Published:** 1906-12-01

**Authors:** 


### SOCIAL AND POOR LAW PROBLEMS.

### WHAT TO DO WITH POOR-LAW BOYS.

A gentleman who is resigning his connection with one of the Metropolitan Boards of Guardians has been pointing out the difficulties which arise in the placing out in life of boys who have been brought up by the poor-law. The law permits of boys being apprenticed to any decent trade, the Guardians paying the premium, but insists that the apprentice should live in his master's house. This regulation is sufficient in itself to keep the boys out of all large businesses, and of many trades altogether. The old idea of apprenticeship is, if not dead, confined to only a few industries, and to the minor practitioners of these. A few small tailors and shoemakers and the lower class of hairdressers are almost the only people who will take poor-law boys as apprentices, and often these are more anxious to secure the boy's premium than his services. In addition there are the *Exmouth* training ship and the army bands. The latter demands some musical faculty, which all boys do not possess, while with regard to the former it is said that many shipowners and masters are not anxious to have the boys after their period of training is over. They prefer to take them direct from home or school, and do the training themselves. "Working boys' homes," while good as a temporary provision, cannot be regarded as a permanent settlement of a boy's future. Many of these difficulties would vanish if it were not for the residential clause in the apprenticeship regulations. If it were permitted to board a boy in one place and let him work in another, it would be much easier than it now is to transform all boys into skilled workmen, who would be, under ordinary circumstances, unlikely to come under the poor-law again. In many cases it would be possible to persuade a mother, who under ordinary circumstances would take her son home from the schools to make him first a message boy, then an unskilled labourer, and, at the end of a comparatively short time, a pauper again, to let her boy be apprenticed to a hopeful trade if he might stay with her on receipt of a very small sum. Of course, such a possibility would depend as much upon the respectability of the mother as on any other factor, but if that be present, this method of providing for the boys would not be more expensive than the present, and would draw in many cases that now escape. Where there are no relations, or where these are undesirable, some extension of the boarding-out system might meet the case. Certainly it would require the consent of the Local Government Board to the subsidising of boys up to eighteen years of age under something other than the apprenticeship system, but this consent is sometimes given, nor is it likely to be refused while Mr. John Burns is at the head of affairs. Let it once be realised that the residential condition of apprenticeship is not suitable to modern conditions, and all the rest will follow.

